# Supplementary material for: Lack of caspase 8 directs neuronal progenitor-like reprogramming and small cell lung cancer progression
Source: Nat Commun. 2025 Dec 18;16:11280. doi: 10.1038/s41467-025-67142-4 (PMC12717127; doi:10.1038/s41467-025-67142-4)
Supplement: Supplementary file 1 — Supplementary Infomation [file 41467_2025_67142_MOESM1_ESM.pdf]

**Supplementary File**

# Lack of Caspase 8 Directs Neuronal Progenitor- like reprogramming and Small Cell Lung Cancer Progression

**Ariadne Androulidaki[1,2], Fanyu Liu[1,2], Christina M. Bebber[1,2], Ilmars Kisis[1], Vignesh Sakthivelu[1], Pascal Hunold[3], Lioba Koerner[2,4], Alina Dahlhaus[1,2], Fatma Isil Yapici[1,2], Christina Grimm[3,5], Alicja Pacholewska[3,5], Sofya Tishina[1,2], Franka Doskotz[1,2], Lucia A. Torres Fernández[1], Jenny Stroh[1,2], Ali T. Abdallah[2,6,7], Julia Beck[1,2], Lejla Mulalic[1,2], Anna Schmitt[2,8], Holger Grüll[9], Thorsten Persigehl[9], Alexander Quaas[10], Martin Peifer[1,3], Johannes Brägelmann[1,3,11], H. Christian Reinhardt[12], Pascal Nieper[1,8], Robert Hänsel-Hertsch[3], Roman K. Thomas[1,10], Julie George[1,13], Michal R. Schweiger[3,5], Manolis Pasparakis[2,3,4], Filippo Beleggia[1,8,11] and Silvia von Karstedt[1,2,3, *]**

[1]University of Cologne, Faculty of Medicine and University Hospital Cologne, Department of Translational Genomics, Cologne, Germany.

[2]CECAD Cluster of Excellence, Faculty of Medicine and University Hospital Cologne, Cologne, Germany.

[3]Center for Molecular Medicine Cologne, Faculty of Medicine and University Hospital Cologne, Cologne, Germany.

[4]University of Cologne, Institute for Genetics, Cologne, Germany

[5]University of Cologne, Institute for Translational Epigenetics, Faculty of Medicine and University Hospital Cologne, Cologne, Germany

[6]Institute of Medical Statistics and Computational Biology, Faculty of Medicine, University of Cologne

[7]CECAD Cluster of Excellence, Faculty of Mathematics and Natural Sciences, University of Cologne

[8]Department I of Internal Medicine, Faculty of Medicine and University Hospital Cologne, Cologne, Germany.

[9]University of Cologne, Department of Radiology, Faculty of Medicine and University Hospital Cologne, Cologne, Germany.
[10]University of Cologne, Institute of Pathology, Faculty of Medicine and University Hospital Cologne, Cologne, Germany.
[11]University of Cologne, Faculty of Medicine and University Hospital Cologne, Mildred Scheel School of Oncology (MSSO), Cologne, Germany
[12]Department of Hematology and Stem Cell Transplantation, University Hospital Essen, University Duisburg-Essen, German Cancer Consortium (DKTK partner site Essen), Essen, Germany.
[13]Department of Otorhinolaryngology, Head and Neck Surgery, Faculty of Medicine and University Hospital Cologne, University Hospital of Cologne

Corresponding author: s.vonkarstedt@uni-koeln.de

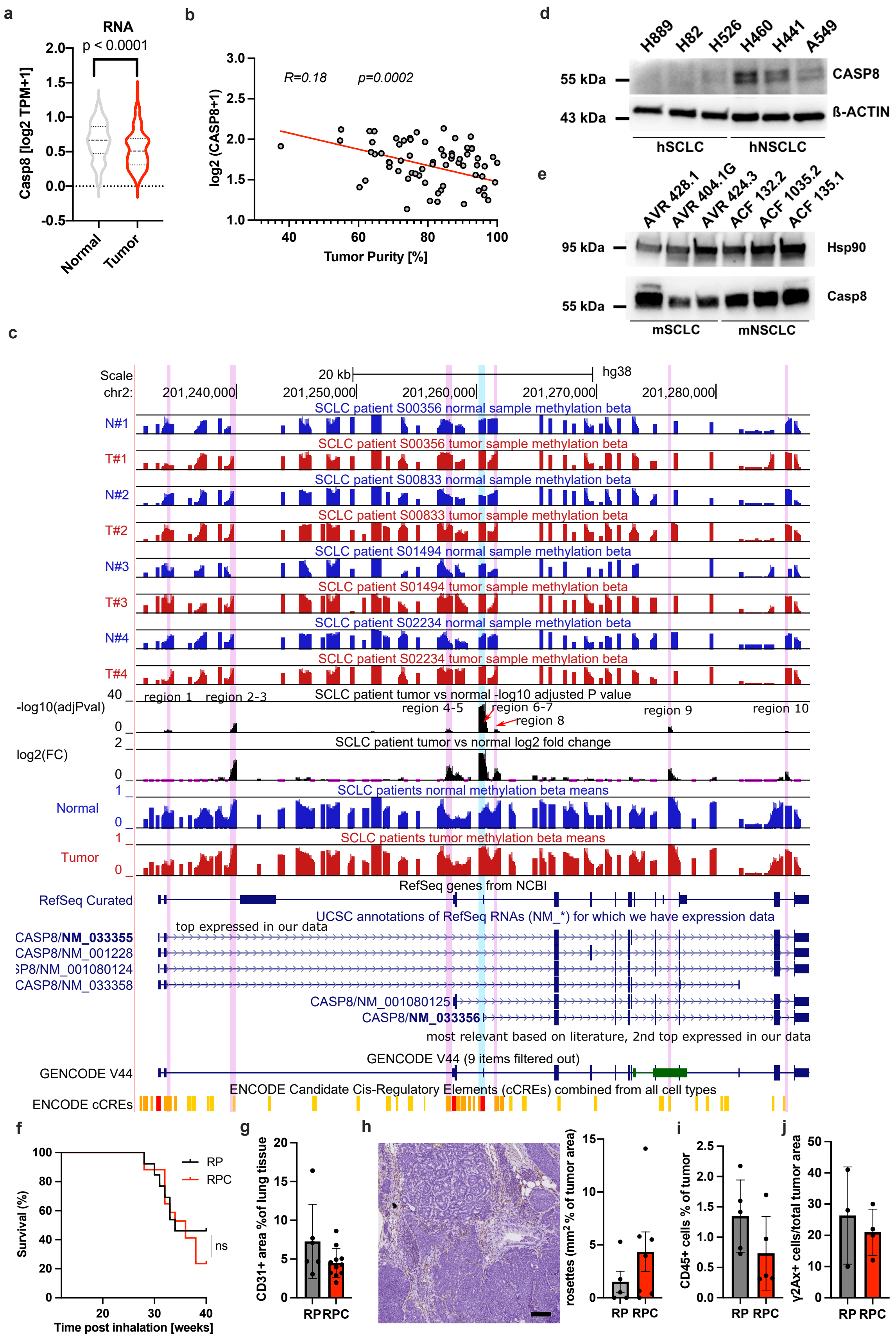

a
RNA
p < 0.0001
Casp8 [log2 TPM+1]
Normal
Tumor
b
R=0.18
p=0.0002
log2 (CASP8+1)
Tumor Purity [%]
d
H889
H82
H526
H460
H441
A549
55 kDa
43 kDa
CASP8
ß-ACTIN
hSCLC
hNSCLC
e
AVR 428.1
AVR 404.1G
AVR 424.3
ACF 132.2
ACF 1035.2
ACF 135.1
95 kDa
55 kDa
Hsp90
Casp8
mSCLC
mNSCLC
c
Scale
chr2:
20 kb
hg38
SCLC patient S00356 normal sample methylation beta
SCLC patient S00356 tumor sample methylation beta
SCLC patient S00833 normal sample methylation beta
SCLC patient S00833 tumor sample methylation beta
SCLC patient S01494 normal sample methylation beta
SCLC patient S01494 tumor sample methylation beta
SCLC patient S02234 normal sample methylation beta
SCLC patient S02234 tumor sample methylation beta
-log10(adjPval)
SCLC patient tumor vs normal -log10 adjusted P value
region 1
region 2-3
region 4-5
region 6-7
region 8
region 9
region 10
log2(FC)
SCLC patient tumor vs normal log2 fold change
SCLC patients normal methylation beta means
SCLC patients tumor methylation beta means
Normal
Tumor
RefSeq Curated
RefSeq genes from NCBI
UCSC annotations of RefSeq RNAs (NM_*) for which we have expression data
top expressed in our data
CASP8/NM_033355
CASP8/NM_001228
CASP8/NM_001080124
CASP8/NM_033358
CASP8/NM_001080125
CASP8/NM_033356
most relevant based on literature, 2nd top expressed in our data
GENCODE V44
GENCODE V44 (9 items filtered out)
ENCODE cCREs
ENCODE Candidate Cis-Regulatory Elements (cCREs) combined from all cell types
f
Survival (%)
RP
RPC
ns
Time post inhalation [weeks]
g
CD31+ area %of lung tissue
RP RPC
h
rosettes (mm² % of tumor area)
RP RPC
i
CD45+ cells % of tumor
RP RPC
j
γ2Ax+ cells/total tumor area
RP RPC

**Supplementary Fig. 1. Lack of Caspase 8 is a feature of human SCLC.**

**a.** Caspase 8 mRNA expression ($log^2$ TPM+1) of SCLC patients (n=112) and matched adjacent normal lung samples (n=112)[1] is plotted. **b.** Expression values for all CASP8 isoforms were summed and correlated to the matching samples with purity information (n=70). Correlation analysis was done with the lm function in R. **c.** Significant differentially methylated regions across the *CASP8* gene. Blue, promoter region of ENST00000323492. N, Normal; T, Tumor. 4 representative patient/matched normal samples out of n=33 are shown. Generation of this panel is further explained in supplementary note 1. **d. e.** Representative immunoblot analysis of protein extracts of (d) human and (e) mouse tumor cell lines showing protein expression of the indicated proteins. **f.** Kaplan-Meier survival of mice after Ad-Cre inhalation (RP n=13 and RPC n=17). **g.** Quantification (qPath) of CD31 positive areas in endpoint tumors of RP (n=6) and RPC (n=11) mice **h.** Representative H&E with rosette area within endpoint tumor. Rosettes areas ($mm^2$) were summed and expressed as % of tumor area per mouse using ImageJ v1.52. Each dot represents a different mouse (RP n=5 and RPC n=7). Scale bars represent 100μm. **i.** Quantification of CD45 positive areas per mouse RP (n=5) and RPC (n=5) mice. **j.** Numbers of phospho γ-2Ax positive cells per total tumor area per mouse. RP (n=3) and RPC (n=4) mice. (d) paired t-test, (f) Log-rank (Mantel-Cox) test (p=0.465). Source Data are provided as a Source Data file.

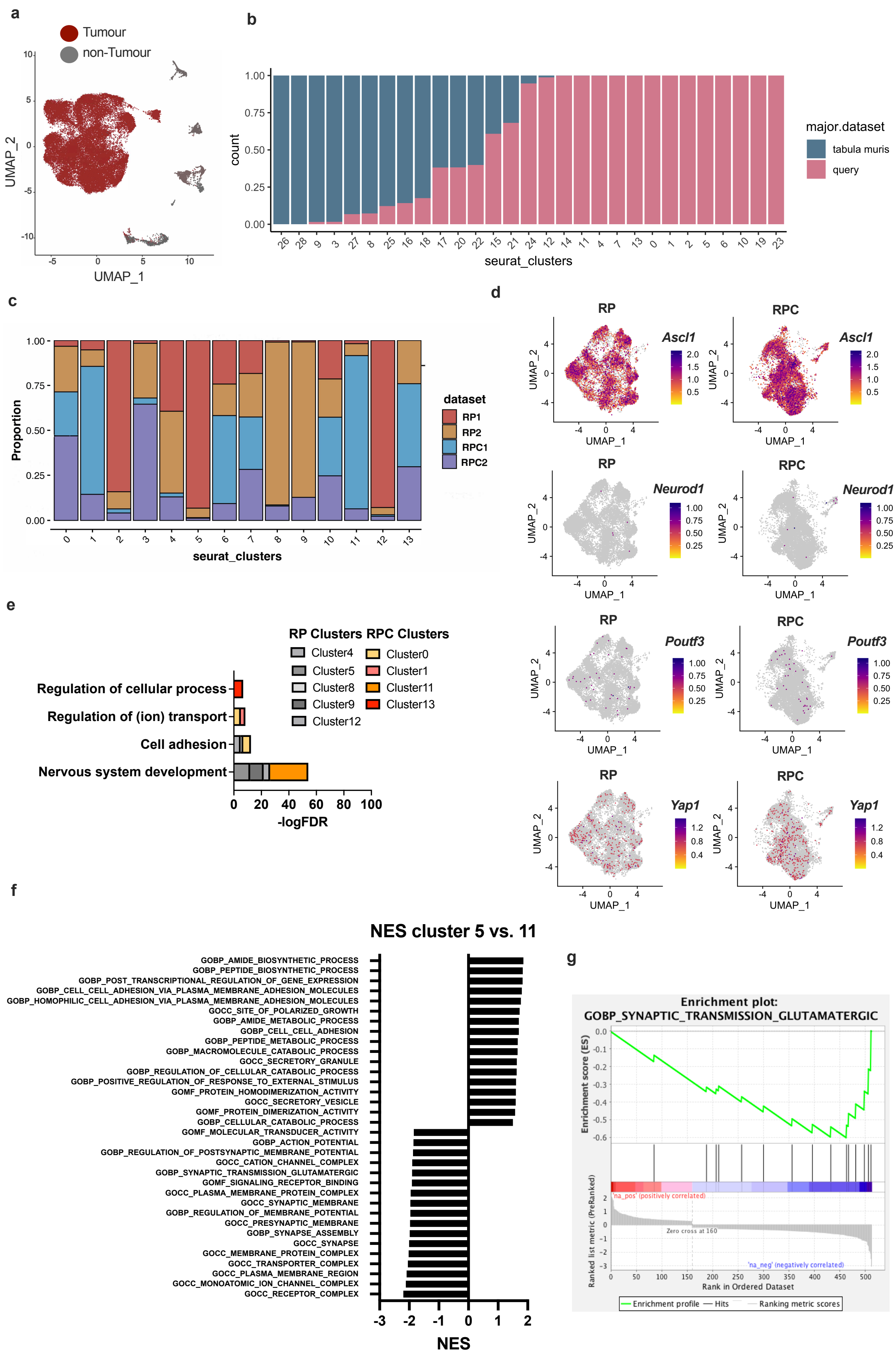
a
Tumour
non-Tumour
UMAP_2
UMAP_1
b
count
major.dataset
tabula muris
query
seurat_clusters
c
Proportion
dataset
RP1
RP2
RPC1
RPC2
seurat_clusters
d
RP
RPC
Ascl1
Neurod1
Poutf3
Yap1
UMAP_1
UMAP_2
e
RP Clusters RPC Clusters
Cluster4
Cluster5
Cluster8
Cluster9
Cluster12
Cluster0
Cluster1
Cluster11
Cluster13
Regulation of cellular process
Regulation of (ion) transport
Cell adhesion
Nervous system development
-logFDR
f
NES cluster 5 vs. 11
GOBP_AMIDE_BIOSYNTHETIC_PROCESS
GOBP_PEPTIDE_BIOSYNTHETIC_PROCESS
GOBP_POST_TRANSCRIPTIONAL_REGULATION_OF_GENE_EXPRESSION
GOBP_CELL_CELL_ADHESION_VIA_PLASMA_MEMBRANE_ADHESION_MOLECULES
GOBP_HOMOPHILIC_CELL_ADHESION_VIA_PLASMA_MEMBRANE_ADHESION_MOLECULES
GOCC_SITE_OF_POLARIZED_GROWTH
GOBP_AMIDE_METABOLIC_PROCESS
GOBP_CELL_CELL_ADHESION
GOBP_PEPTIDE_METABOLIC_PROCESS
GOBP_MACROMOLECULE_CATABOLIC_PROCESS
GOCC_SECRETORY_GRANULE
GOBP_REGULATION_OF_CELLULAR_CATABOLIC_PROCESS
GOBP_POSITIVE_REGULATION_OF_RESPONSE_TO_EXTERNAL_STIMULUS
GOMF_PROTEIN_HOMODIMERIZATION_ACTIVITY
GOCC_SECRETORY_VESICLE
GOMF_PROTEIN_DIMERIZATION_ACTIVITY
GOBP_CELLULAR_CATABOLIC_PROCESS
GOMF_MOLECULAR_TRANSDUCER_ACTIVITY
GOBP_ACTION_POTENTIAL
GOBP_REGULATION_OF_POSTSYNAPTIC_MEMBRANE_POTENTIAL
GOCC_CATION_CHANNEL_COMPLEX
GOBP_SYNAPTIC_TRANSMISSION_GLUTAMATERGIC
GOMF_SIGNALING_RECEPTOR_BINDING
GOCC_PLASMA_MEMBRANE_PROTEIN_COMPLEX
GOCC_SYNAPTIC_MEMBRANE
GOBP_REGULATION_OF_MEMBRANE_POTENTIAL
GOCC_PRESYNAPTIC_MEMBRANE
GOBP_SYNAPSE_ASSEMBLY
GOCC_SYNAPSE
GOCC_MEMBRANE_PROTEIN_COMPLEX
GOCC_TRANSPORTER_COMPLEX
GOCC_PLASMA_MEMBRANE_REGION
GOCC_MONOATOMIC_ION_CHANNEL_COMPLEX
GOCC_RECEPTOR_COMPLEX
NES
g
Enrichment plot:
GOBP_SYNAPTIC_TRANSMISSION_GLUTAMATERGIC
Enrichment score (ES)
'na_pos' (positively correlated)
Zero cross at 160
'na_neg' (negatively correlated)
Ranked list metric (PreRanked)
Rank in Ordered Dataset
Enrichment profile
Hits
Ranking metric scores

**Supplementary Fig. 2. Caspase 8 deletion promotes neuronal-like reprogramming in SCLC.** **a.** Samples were mapped to the annotated single-cell reference dataset (PMID: 30283141) containing healthy C57BL/6 mouse lung and trachea cells. **b.** Clusters filled predominantly (threshold 95%) by query samples were assigned as tumor cells **c.** Percentages of RP and RPC cells in each cluster separated by single samples (RP1, RP2, RPC1, RPC2). **d.** UMAP projection of SCLC-A/N/P/Y subtype expression of markers within tumor cells. **e.** STRING analysis was performed on the list of upregulated cluster- defining genes for each cluster from scRNA-seq data from RP and RPC mice**.** Representative enriched GO terms within the top 10 ´Biological Process´ pathways are plotted based on -$log^2$ False Discovery Rate (FDR). **f, g.** GSEA of a ranked list between differentially expression genes comparing cluster 5 (RP) with cluster 11 (RPC). Negative enrichment score is plotted (NES). Source Data are provided as a Source Data file.

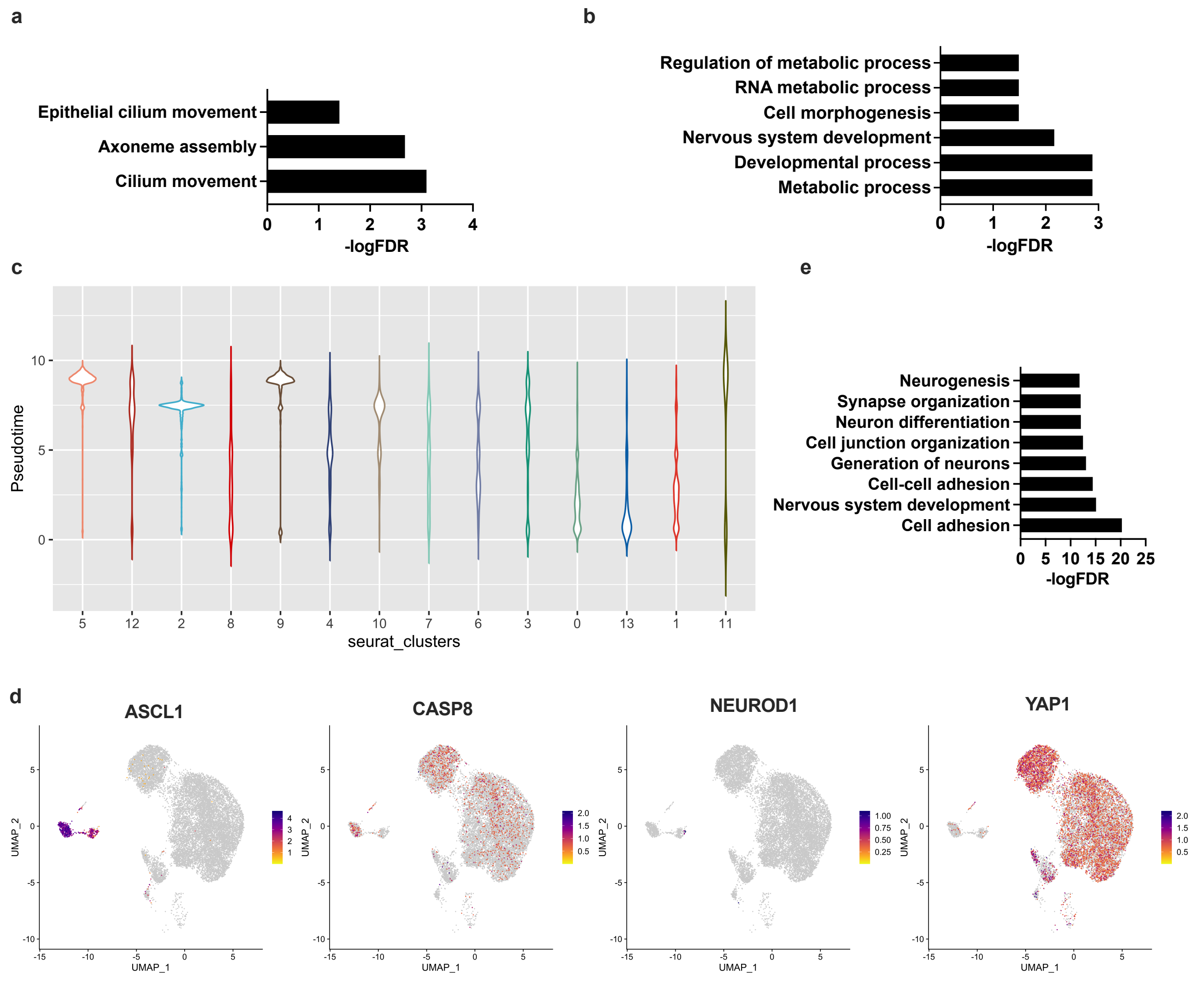


**Supplementary Fig. 3. Deletion of caspase 8 promotes reprogramming towards a neuronal progenitor-like state.**

**a.** Significantly upregulated genes (scRNA-seq pseudobulk) in RPC as compared to RP tumors were subjected to STRING analysis for pathway enrichment. Significantly enriched pathways (GO term ´Biological process´) are shown. -log$^2$ false discovery rate (FDR) is plotted. **b.** Significantly downregulated genes (scRNA-seq pseudobulk) in RPC as compared to RP tumors were subjected to STRING analysis for pathway enrichment. Representative significantly enriched pathways (GO term ´Biological process´) are shown. -log$^2$ false discovery rate (FDR) is plotted. **c.** The 13 tumor clusters were depicted in pseudotime. **d.** Single cell RNA-seq data from isolated RPM cells after day 4, 7, 11, 14, 17 and 21 *ex vivo*[2] were plotted for expression of the indicated genes. **e.** The 200 genes determining the unsupervised pseudotime trajectory were subjected to STRING analysis.

The top 8 (based on false discovery rate) significantly enriched pathways (GO term ´Biological process´) are shown. -$log^2$ FDR is plotted. Source Data are provided as a Source Data file.

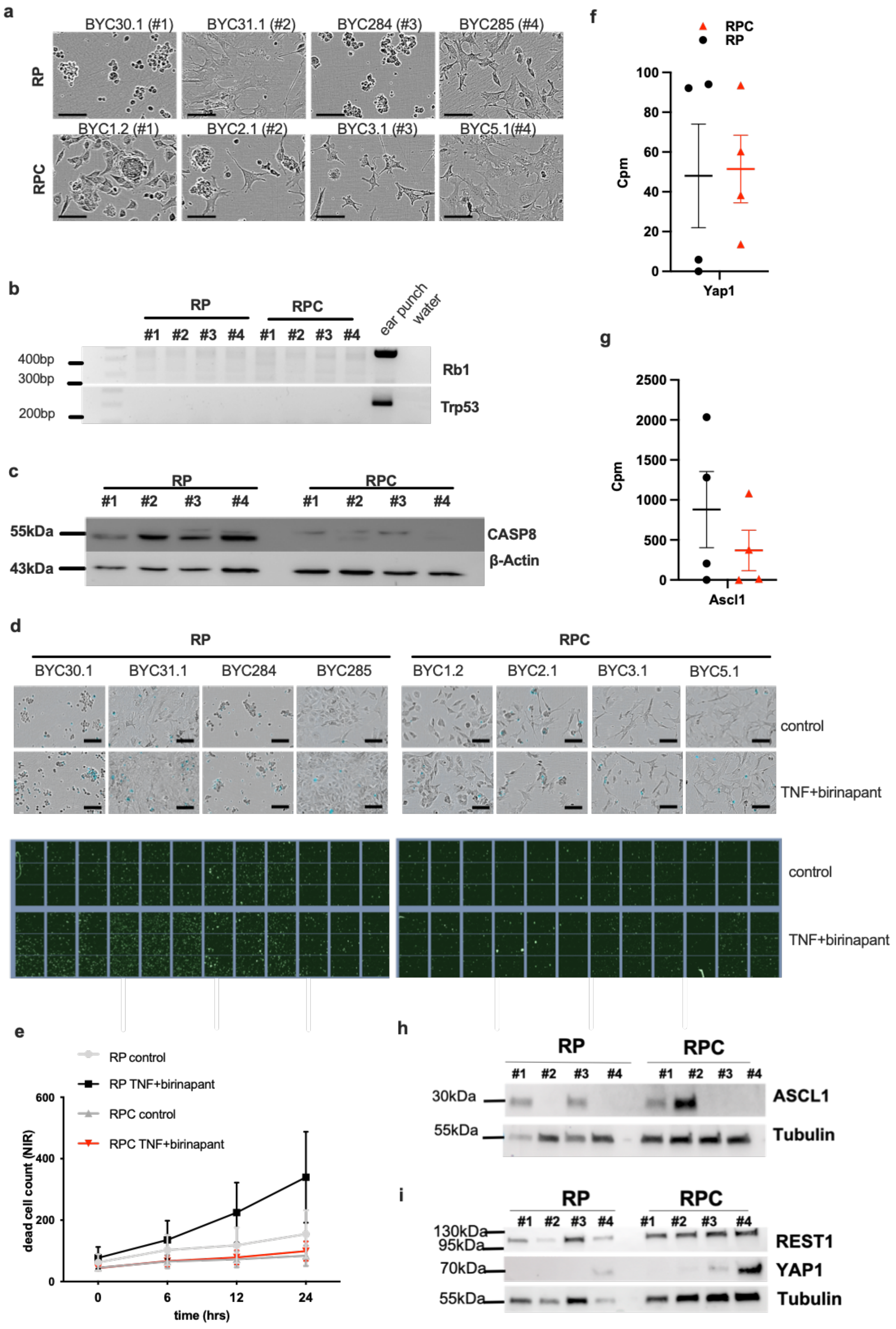
a
BYC30.1 (#1)
BYC31.1 (#2)
BYC284 (#3)
BYC285 (#4)
RP
BYC1.2 (#1)
BYC2.1 (#2)
BYC3.1 (#3)
BYC5.1(#4)
RPC
b
RP
RPC
#1 #2 #3 #4 #1 #2 #3 #4
ear punch
water
400bp
300bp
200bp
Rb1
Trp53
c
RP
RPC
#1 #2 #3 #4 #1 #2 #3 #4
55kDa
43kDa
CASP8
β-Actin
d
RP
RPC
BYC30.1
BYC31.1
BYC284
BYC285
BYC1.2
BYC2.1
BYC3.1
BYC5.1
control
TNF+birinapant
control
TNF+birinapant
e
RP control
RP TNF+birinapant
RPC control
RPC TNF+birinapant
dead cell count (NIR)
time (hrs)
f
RPC
RP
Cpm
Yap1
g
Cpm
Ascl1
h
RP
RPC
#1 #2 #3 #4 #1 #2 #3 #4
30kDa
55kDa
ASCL1
Tubulin
i
RP
RPC
#1 #2 #3 #4 #1 #2 #3 #4
130kDa
95kDa
70kDa
55kDa
REST1
YAP1
Tubulin

**Supplementary Fig. 4. Characterization of RP and RPC cell lines.**

**a.** Representative brightfield images (10x) of cell lines generated from individual endpoint tumors from RP (n=4) and RPC (n=4) mice. Scale bars represent 100µm **b.** Representative PCR analysis of isolated DNA from tumor cell lines confirming deletion of *Rb1* and *Trp53* genes. **c.** Representative immunoblot analysis of protein extracts from RP and RPC tumor cell lines. The indicated proteins were detected. **d.** Live cell imaging was performed on tumor cell lines stimulated with control or TNF [20ng/ml] + birinapant [1µM] in the presence of DRAQ7 [100nM] using the IncuCyte SX5 live cell imaging system (Incucyte). Cell death was determined by DRAQ7 (Biolegend) fluorescence (near infrared, NIR) count. Activation of cleaved caspase 3 was determined by adding a fluorogenic substrate for activated caspase3/7 (CellEvent$^{TM}$ Caspase-3/7 Green Detection Reagent, Invitrogen) [5µM]. Representative brightfield (scale bars 100µm) and fluorescent images (10x) 24 h after stimulation are shown. **e.** Automated quantification of cell death (NIR count) over time is shown Data represent means of three independent experiments +/- SEM. **f, g.** Expression data from the RNAseq analysis (Cpm values) for *Ascl1* and *Yap1* genes. **h, i.** Representative immunoblot analysis of protein extracts from RP and RPC tumor cell lines. The indicated proteins were detected. The samples RP (#1- #4) and RPC (#1- #4) (**c, h, i**) derive from the same experiment but different gels were loaded to detect caspase 8, another for ASCL1, and another for REST1 and YAP1. Separate loading controls are shown in all three cases. Source Data are provided as a Source Data file.

a

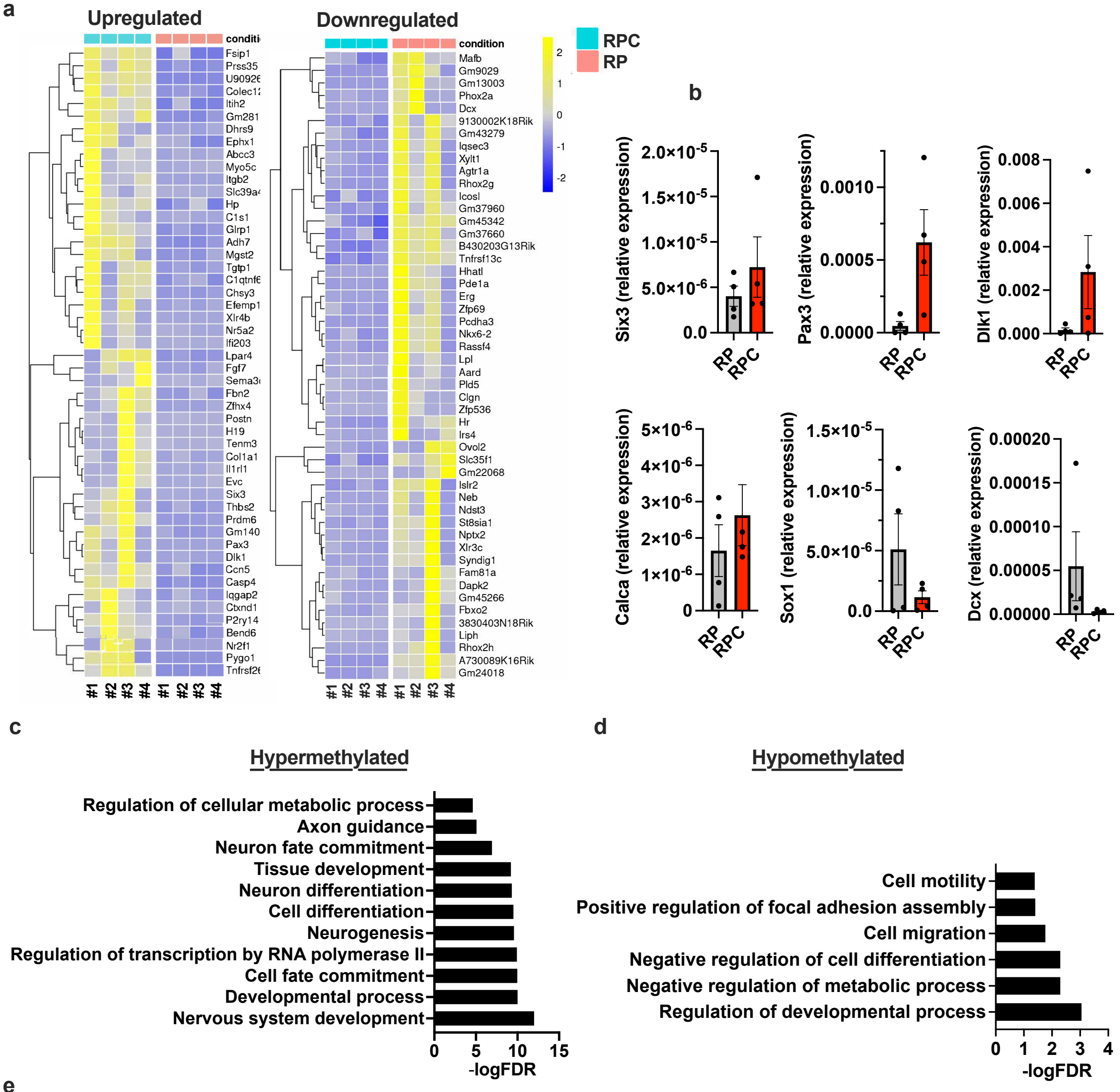


e

Upregulated

| Gene set name (KEGG) | p_value |
|---|---|
| Cell adhesion molecules | 0,012154686 |
| Amoebiasis | 0,048265692 |
| **ECM-receptor interaction** | 0,048265692 |
| Regulation of actin cytoskeleton | 0,067144597 |
| Leukocyte transendothelial migration | 0,067144597 |
| **Focal adhesion** | 0,085163698 |

Hypormethylated

| Gene set name (KEGG) | p_value |
|---|---|
| **Focal adhesion** | 1.3e−01 |
| Neuroactive ligand−receptor interaction | 1.0e+00 |
| **ECM−receptor interaction** | 5.0e−01 |
| PI3K−Akt signaling pathway | 1.0e+00 |
| cAMP signaling pathway | 1.0e+00 |
| Fluid shear stress and atherosclerosis | 3.0e−02 |
| VEGF signaling pathway | 8.4e−03 |
| EGFR tyrosine kinase inhibitor resistance | 2.8e−02 |

f

Downregulated

| Gene set name (BP) | p_value |
|---|---|
| **developmental process** | 0,002702696 |
| **cell differentiation** | 0,004580482 |
| **cellular developmental process** | 0,004580482 |
| **anatomical structure development** | 0,004580482 |
| cell development | 0,01266037 |
| **neuron differentiation** | 0,018375491 |
| nervous system development | 0,018375491 |
| **system development** | 0,018375491 |
| **multicellular organism development** | 0,018375491 |
| cellular response to stimulus | 0,024849065 |
| neurogenesis | 0,024849065 |

Hypermethylated

| Gene set name (BP) | p_value |
|---|---|
| **system development** | 7.8e−16 |
| **multicellular organism development** | 1.3e−14 |
| anatomical structure morphogenesis | 1.3e−12 |
| **anatomical structure development** | 1.0e−12 |
| animal organ development | 1.0e−10 |
| **developmental process** | 1.2e−11 |
| negative regulation of cellular process | 1.5e−09 |
| regulation of cellular process | 1.5e−12 |
| **cellular developmental process** | 1.1e−12 |
| **cell differentiation** | 1.9e−12 |
| **neuron differentiation** | 1.5e−15 |

**Supplementary Fig. 5. RPC cell lines retain the neuronal progenitor-like phenotype.**

**a.** RPC-derived cell lines (n=4, red) as compared to RP-derived cell lines (n=4, blue) were subjected to bulk RNA-seq. Heat maps of the top 50 significantly up- and down- regulated genes are shown. **b.** qPCR analysis comparing gene expression between RP- (n=4, grey) and RPC- (n=4, red) derived cell lines. Relative expression compared to the housekeeping gene *Actin* is shown. **c.** Significantly hypermethylated genes in RPC-derived cells were subjected to STRING analysis for pathway enrichment. Representatives of the top significantly enriched pathways (GO term ´Biological process´) are shown. $-log^2$ false discovery rate (FDR) is plotted. **d.** Significantly hypomethylated genes in RPC-derived cells were subjected to STRING analysis for pathway enrichment. Representatives of the significantly enriched pathways (GO term ´Biological process´) are shown. $-log^2$ false discovery rate (FDR) is plotted. **e.** GO term enrichment analysis of the significantly upregulated genes (left table) and g:Profiler analysis of the significantly hypomethylated genes (right table) in RPC cell lines (GO term ´KEGG´). **f.** GO term enrichment analysis of the significantly downregulated genes (left table) and g:Profiler analysis of the significantly hypermethylated genes (right table) in RPC cell lines (GO term ´Biological Process´). Source Data are provided as a Source Data file.

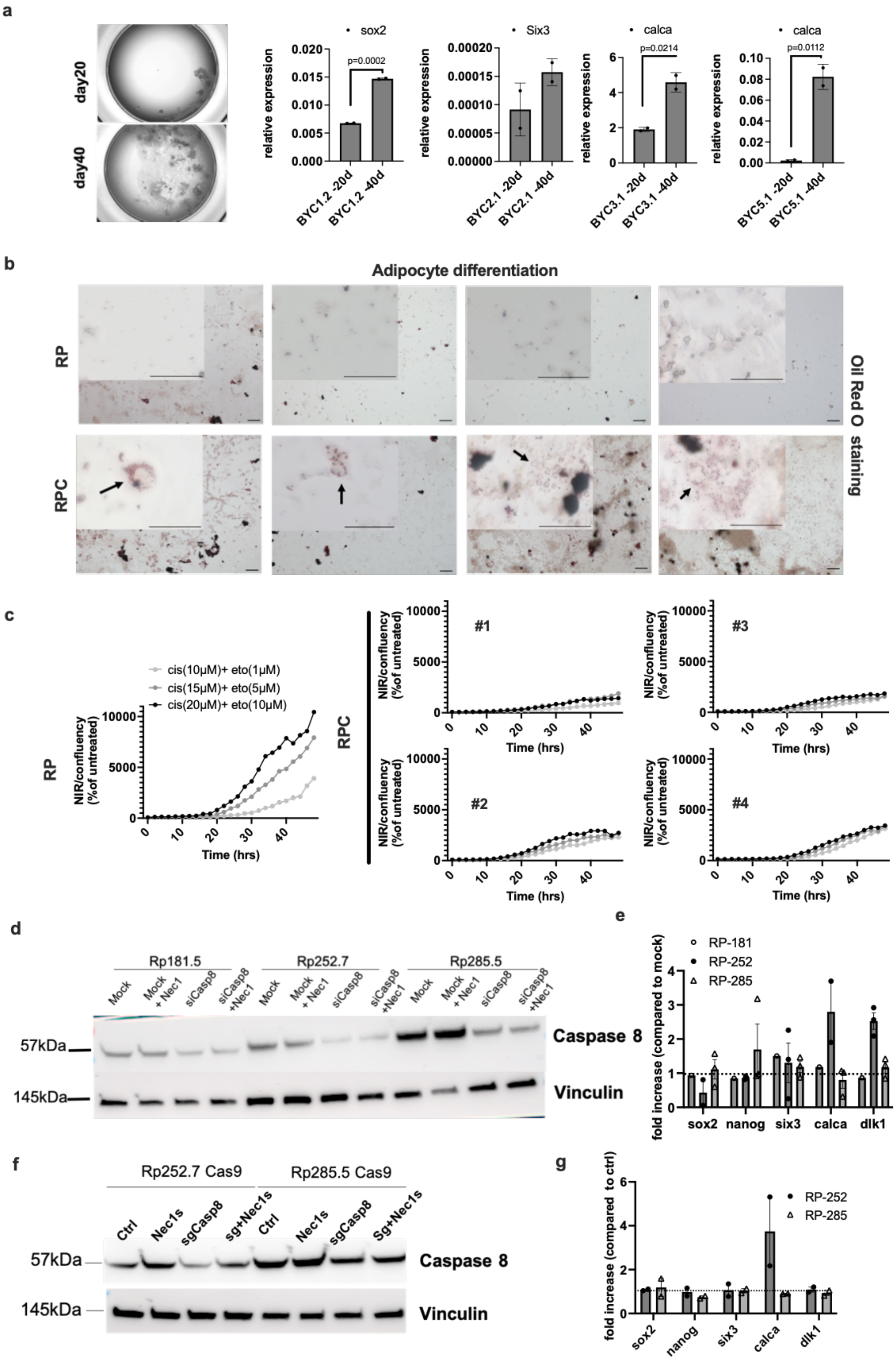
a
day20
day40
sox2
Six3
calca
calca
relative expression
p=0.0002
p=0.0214
p=0.0112
BYC1.2 -20d
BYC1.2 -40d
BYC2.1 -20d
BYC2.1 -40d
BYC3.1 -20d
BYC3.1 -40d
BYC5.1 -20d
BYC5.1 -40d
b
Adipocyte differentiation
RP
RPC
Oil Red O staining
c
cis(10μM)+ eto(1μM)
cis(15μM)+ eto(5μM)
cis(20μM)+ eto(10μM)
RP
RPC
NIR/confluency (%of untreated)
Time (hrs)
#1
#2
#3
#4
d
Rp181.5
Rp252.7
Rp285.5
Mock
Mock + Nec1
siCasp8
siCasp8 +Nec1
57kDa
145kDa
Caspase 8
Vinculin
e
RP-181
RP-252
RP-285
fold increase (compared to mock)
sox2
nanog
six3
calca
dlk1
f
Rp252.7 Cas9
Rp285.5 Cas9
Ctrl
Nec1s
sgCasp8
sg+Nec1s
Sg+Nec1s
57kDa
145kDa
Caspase 8
Vinculin
g
RP-252
RP-285
fold increase (compared to ctrl)
sox2
nanog
six3
calca
dlk1

**Supplementary Fig. 6. RPC cell lines show features of pluripotency while caspase 8 silencing *ex vivo* is insufficient to induce expression of progenitor cell markers.**

**a.** RPC cells were seeded in low adhesion plates for spheroid formation assays. Cells were collected at days 20 and 40 and analyzed by qPCR for the indicated genes. Data represent means of two independent experiments for each depicted cell line. Photos are representative of cells at day 20 and at day 40. **b.** RP (n=4) and RPC (n=4) cell lines were cultured for 3 weeks in adipocyte differentiation media (Thermofisher) according to the manufacturer's instructions and subsequently stained with Oil Red O. Arrows indicate characteristic lipid droplet staining. Scale bars represent 100µm **c.** In vivo live imaging (Incucyte) of RP and RPC cell lines after treatment with cisplatin plus etoposide for the indicated doses and time points. Data represent cell death (NIR count) normalized to confluency and are means of 3 replicates per cell line. **d.** Expression of caspase 8 protein levels after ex vivo si-RNA-mediated silencing in three different RP-derived cell lines (+/- nec1s [10µM]) and **e.** qPCR expression analysis of neuronal progenitor marker genes after caspase 8 silencing. Data (relative expression) are presented as fold increase compared to mock. For each cell line data represent means of 3 independent experiments. **f.** Expression of caspase 8 protein levels after ex vivo CRISPR/Cas9-mediated knockout in two different Cas9-expressing RP-derived cell lines (+/- nec1s [10µM]) and **g.** qPCR expression analysis of neuronal progenitor marker genes after caspase 8 knockdown. Data are presented as fold increase compared to control. For each cell line data represent means of 2 independent experiments. Data are plotted +/- SEM throughout. **a.** two-tailed unpaired *t*-tests. Source Data are provided as a Source Data file.

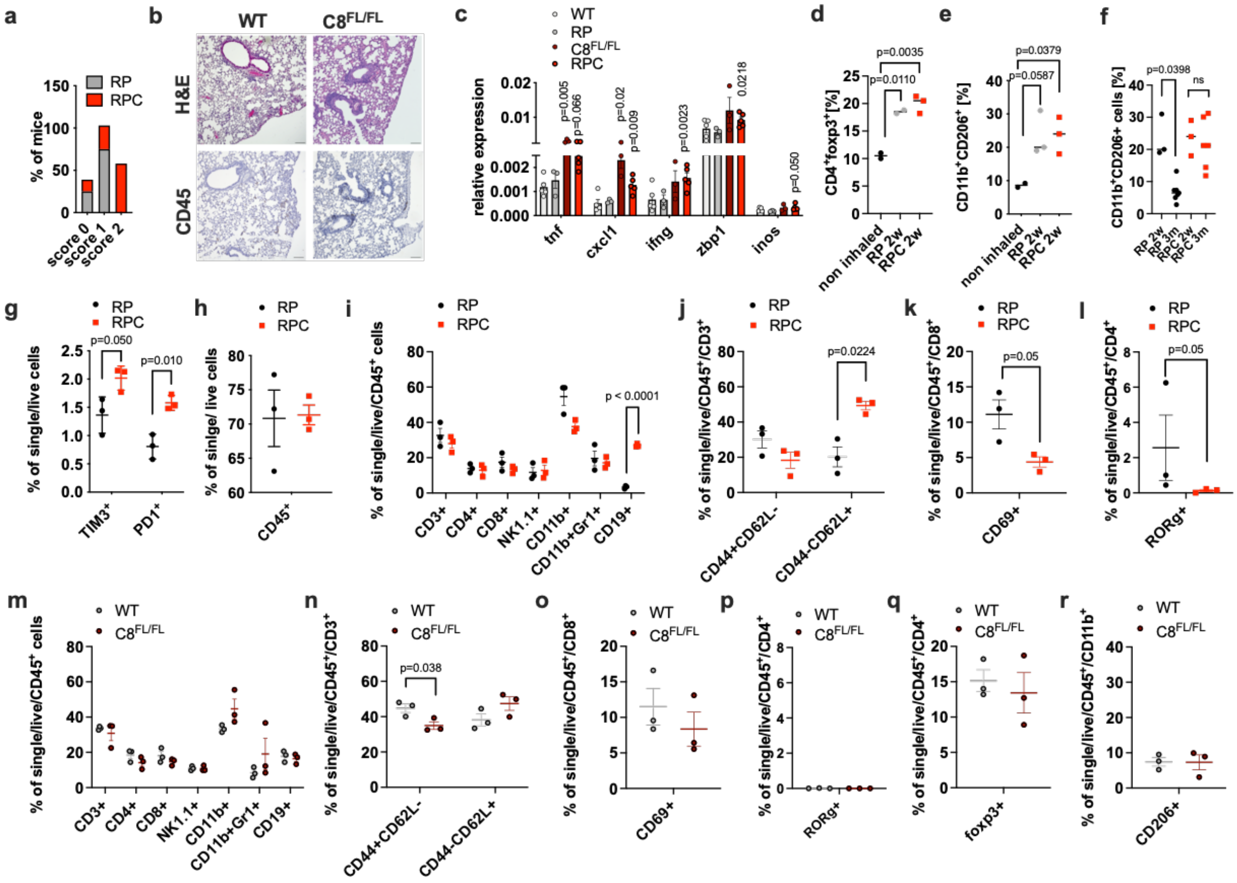

**Supplementary Fig. 7. Caspasse 8 deletion results in persistent immunosuppression only in the context of *Trp53*/*Rb1* co-deletion.**

**a.** Score of peri-bronchial infiltrates after macroscopical examination of CD45-stained lung sections from RP (n=4) and RPC (n=7) mice at 2 weeks post inhalation (0: no infiltrates, score 1: mild peri-bronchial infiltrates, score 2: extended peri-bronchial infiltration) was quantified by pathological inspection. **b.** Representative H&E and CD45 stained 10x images of WT (n=3) and $C8^{FL/FL}$ (n=3) mice (scale bar 10µm). **c.** qPCR analysis of whole lungs of WT (n=5), $C8^{FL/FL}$ (n=3), RP (n=3) and RPC (n=5) mice 2 weeks post Ad-Cre inhalations. Data are presented as relative expression compared to the housekeeping gene *Actin*. p values between WT and $C8^{FL/FL}$, or, RP and RPC are depicted. **d, e.** At two weeks post Ad-Cre inhalation, immune cells from whole lungs from RP (n=3) and RPC (n=3) mice or non-inhaled mice (n=2) were analysed by FACS for Tregs (d) and M2 macrophages (e). Proportions are calculated within $CD45^+$ cells. **f.** Comparison of the proportions of M2 macrophages between 2 weeks and 3 months post inhalation in RP and RPC

mice. **g-l.** FACS analysis of RP (n=3) and RPC (n=3) or **m-r.** C8$^{FL/FL}$ (n=3) and WT (n=3) whole lungs at 12 weeks post inhalation for the indicated immune populations. **d-r.** two-tailed unpaired t-tests with Welch´s correction. Data are presented +/- SEM. Source Data are provided as a Source Data file.

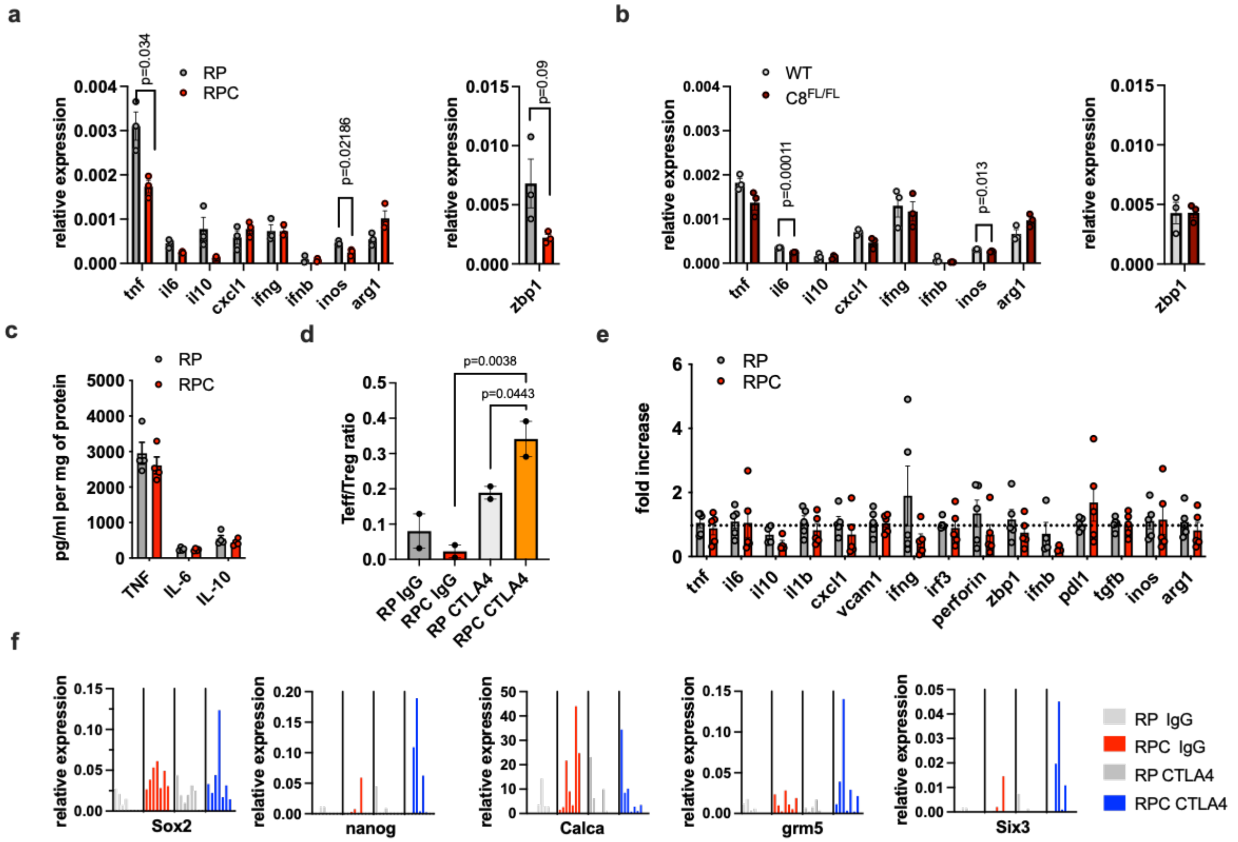


**Supplementary Fig. 8. Time-dependent changes in inflammatory cytokine expression.**

**a, b.** qPCR analysis 12 weeks post Ad-Cre inhalation (RP n=3, RPC n=3, C8$^{FL/FL}$=3, WT=3). Expression data were normalised to the housekeeping gene *Actin* **c.** TNF, IL-6 and IL-10 levels from whole lungs of RP (n=4) and RPC (n=4) mice 18 weeks post Ad-Cre inhalation were quantified using ELISA **d.** 12 weeks post Ad-Cre inhalation, RP and RPC mice were treated intraperitonially either with IgG [5mg/kg] or anti-CTLA-4 [5mg/kg] every other day for 2 weeks. Two mice per genotype and per condition were taken 48hrs after the completion of the treatment and whole lungs were analysed by FACS for effector T cell (CD8$^{+}$) and Treg proportions.

Teff/Treg ratios are depicted. **e.** qPCR analysis of whole lungs of RP and RPC mice at humane endpoint. Data were normalised to the housekeeping gene *Actin* and expressed as fold increase compared to expression in RP mice (n=5 per genotype). Dots represent individual mice. **f.** qPCR gene expression analysis in endpoint tumors of mice treated with IgG or CTLA-4. RP IgG (n=4), RPC IgG (n=7), RP CTLA-4 (n=6) and RPC CTLA-4 (n=7). Relative expression to housekeeping gene *Actin* is plotted. Data are presented +/- SEM. two-tailed unpaired *t* tests. Source Data are provided as a Source Data file.

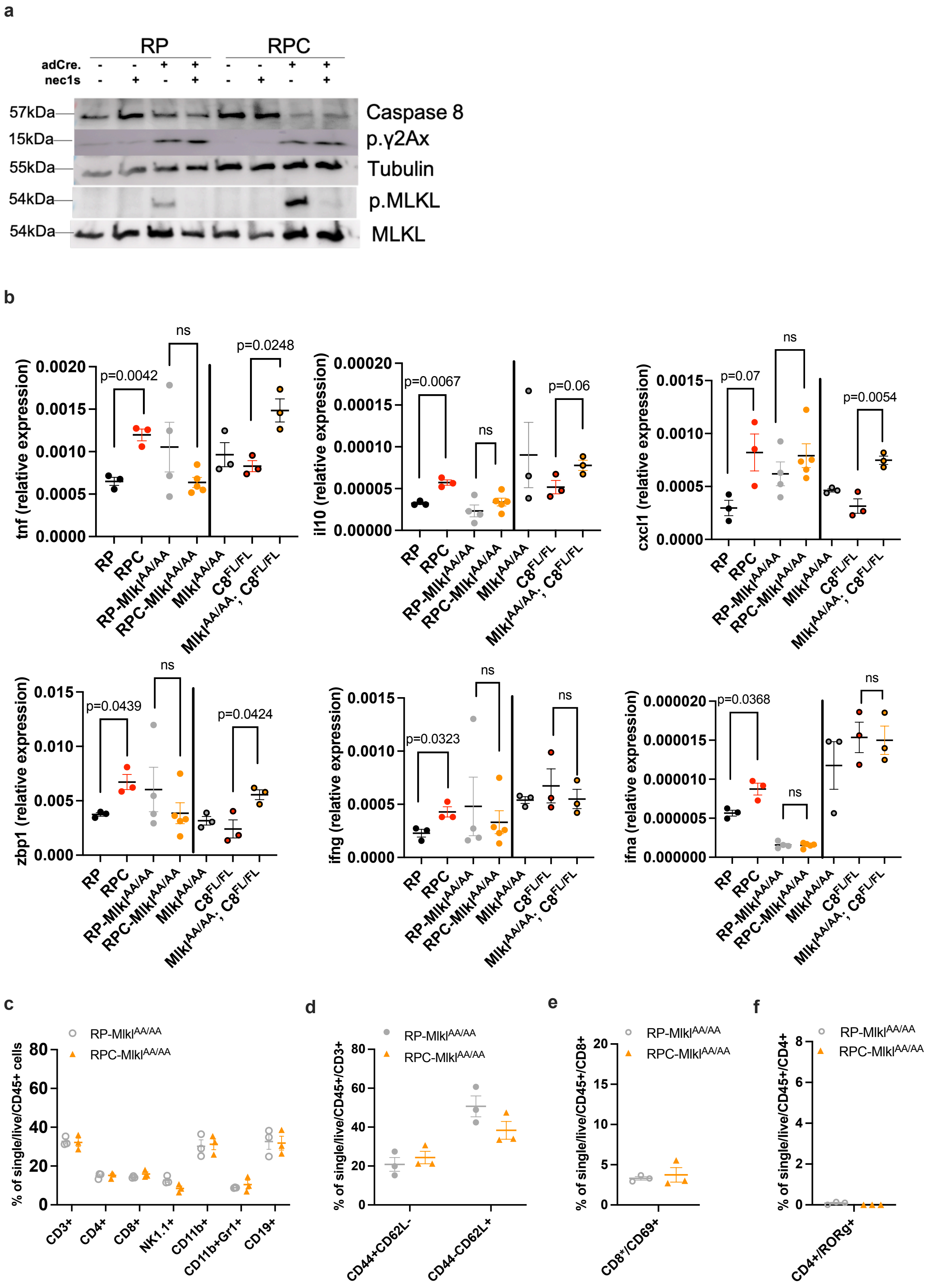
a
RP
RPC
adCre.
nec1s
57kDa
15kDa
55kDa
54kDa
54kDa
Caspase 8
p.γ2Ax
Tubulin
p.MLKL
MLKL
b
tnf (relative expression)
il10 (relative expression)
cxcl1 (relative expression)
zbp1 (relative expression)
ifng (relative expression)
ifna (relative expression)
p=0.0042
p=0.0248
p=0.0067
p=0.06
p=0.07
p=0.0054
p=0.0439
p=0.0424
p=0.0323
p=0.0368
ns
RP
RPC
RP-Mlkl^AA/AA
RPC-Mlkl^AA/AA
Mlkl^AA/AA
C8^FL/FL
Mlkl^AA/AA; C8^FL/FL
c
d
e
f
RP-Mlkl^AA/AA
RPC-Mlkl^AA/AA
% of single/live/CD45+ cells
% of single/live/CD45+/CD3+
% of single/live/CD45+/CD8+
% of single/live/CD45+/CD4+
CD3+
CD4+
CD8+
NK1.1+
CD11b+
CD11b+Gr1+
CD19+
CD44+CD62L-
CD44-CD62L+
CD8+/CD69+
CD4+/RORg+

**Supplementary Fig. 9. Necroptosis promotes pre- tumoral inflammation.**

**a.** Immunoblot analysis of primary lung fibroblasts from RP and RPC mice 48 hours after *in vitro* treatment with AdCre, in the presence or not of nec1s (10µM), for the indicated proteins. **b.** Expression of inflammatory genes in the lungs of the indicated genotypes at 5 days post inhalation by qPCR analysis. Each dot represents a mouse. Data are presented as relative expression compared to the housekeeping gene *Actin*. **c- f.** FACS analysis of RP-Mlkl$^{AA/AA}$ (n=3) and RPC-Mlkl$^{AA/AA}$ (n=3) whole lungs at 12 weeks post inhalation for the indicated immune populations. **b.** unpaired t-tests with Welch´s correction. Data are presented +/- SEM. Source Data are provided as a Source Data file.

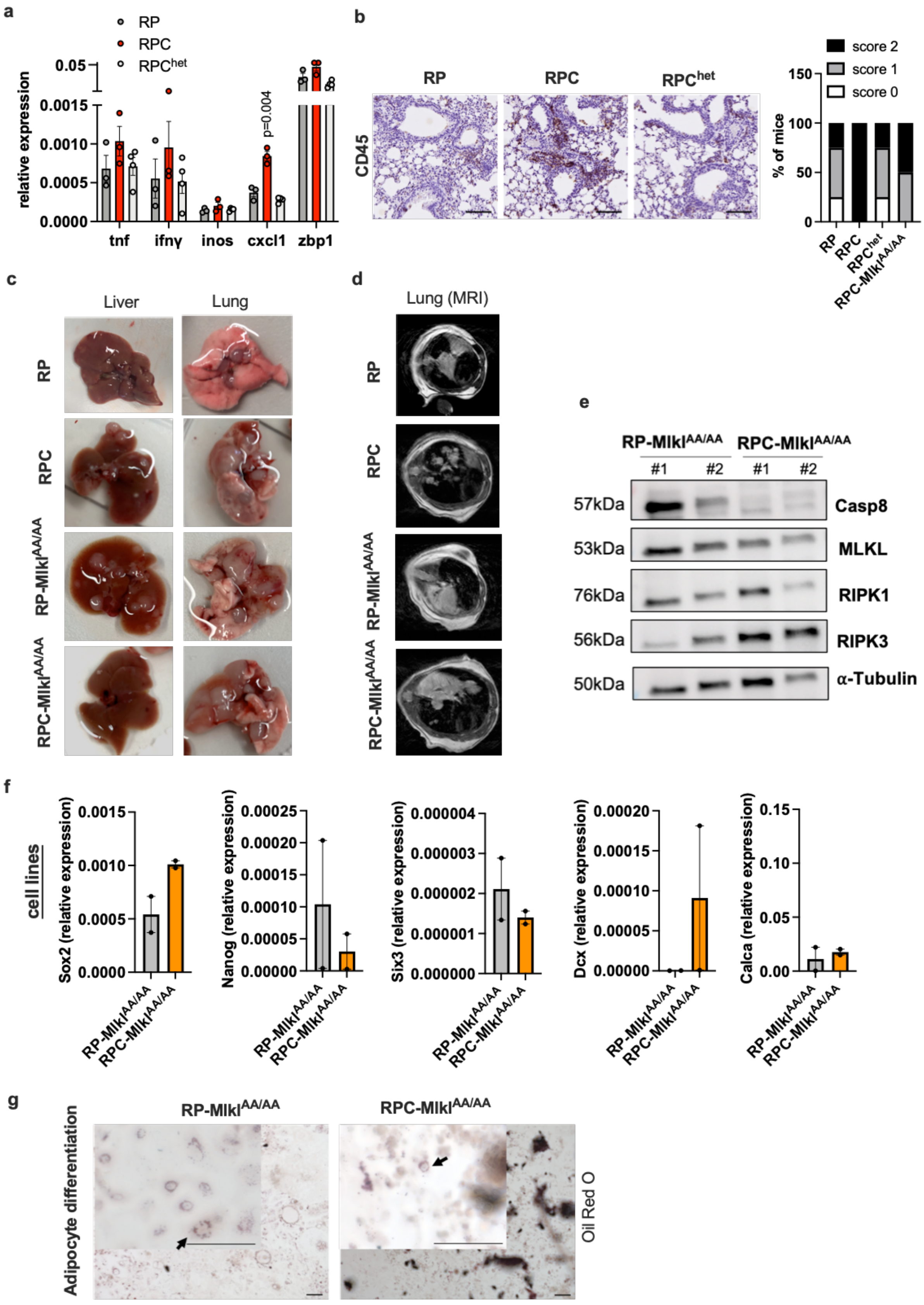
a
RP
RPC
RPChet
relative expression
0.05
0.0015
0.0010
0.0005
0.0000
p=0.004
tnf
ifnγ
inos
cxcl1
zbp1
b
RP
RPC
RPChet
CD45
score 2
score 1
score 0
% of mice
150
100
50
0
RP
RPC
RPChet
RPC-MlklAA/AA
c
Liver
Lung
RP
RPC
RP-MlklAA/AA
RPC-MlklAA/AA
d
Lung (MRI)
RP
RPC
RP-MlklAA/AA
RPC-MlklAA/AA
e
RP-MlklAA/AA
RPC-MlklAA/AA
#1
#2
#1
#2
57kDa
Casp8
53kDa
MLKL
76kDa
RIPK1
56kDa
RIPK3
50kDa
α-Tubulin
f
cell lines
Sox2 (relative expression)
0.0015
0.0010
0.0005
0.0000
Nanog (relative expression)
0.00025
0.00020
0.00015
0.00010
0.00005
0.00000
Six3 (relative expression)
0.000004
0.000003
0.000002
0.000001
0.000000
Dcx (relative expression)
0.00020
0.00015
0.00010
0.00005
0.00000
Calca (relative expression)
0.15
0.10
0.05
0.00
RP-MlklAA/AA
RPC-MlklAA/AA
g
RP-MlklAA/AA
RPC-MlklAA/AA
Adipocyte differentiation
Oil Red O

**Supplementary Fig. 10. Necroptosis promotes the establishment of inflammation, metastasis and features of stemness in RPC mice.**

**a.** qPCR analysis of whole lungs of RP, RPC and $RPC^{het}$ mice 2 weeks post Ad-Cre inhalations. Data are presented as relative expression compared to the housekeeping gene *Actin*. **b.** CD45 immunohistochemistry staining and quantification of the indicated genotypes 2 weeks post Ad-Cre inhalation. RP (n=3), RPC (n=3) and $RPC^{het}$ (n= 4). Representative images are shown, scale bars represent 100µm. Score of peri-bronchial infiltrates after macroscopical examination of CD45-stained lung sections (0: no infiltrates, score 1: mild peri-bronchial infiltrates, score 2: extended peri-bronchial infiltration) was quantified by pathological inspection. **c.** Representative photos of lung tumors and liver metastasis at humane endpoint. **d.** Representative MRI images at 30 weeks post Ad-Cre inhalation **e.** Immunoblot analysis of the indicated proteins in RP-$Mlkl^{AA/AA}$ (n=2) and RPC-$Mlkl^{AA/AA}$ (n=2) cell lines. **f.** qPCR analysis comparing gene expression between RP-$Mlkl^{AA/AA}$ (n=2) and RPC-$Mlkl^{AA/AA}$ (n=2) cell lines. Relative expression compared to the housekeeping gene *Actin* is plotted. Dots represent individual mice or cell lines. **g.** RP-$Mlkl^{AA/AA}$ (n=2) and RPC-$Mlkl^{AA/AA}$ (n=2) cell lines were cultured for 3 weeks in adipocyte differentiation media (Thermofisher) according to manufacture instructions and subsequently stained with Oil Red O. Characteristic lipid droplet staining is indicated by arrows in both genotypes. Scale bars represent 100µm. Data are presented +/- SEM. Source Data are provided as a Source Data file.

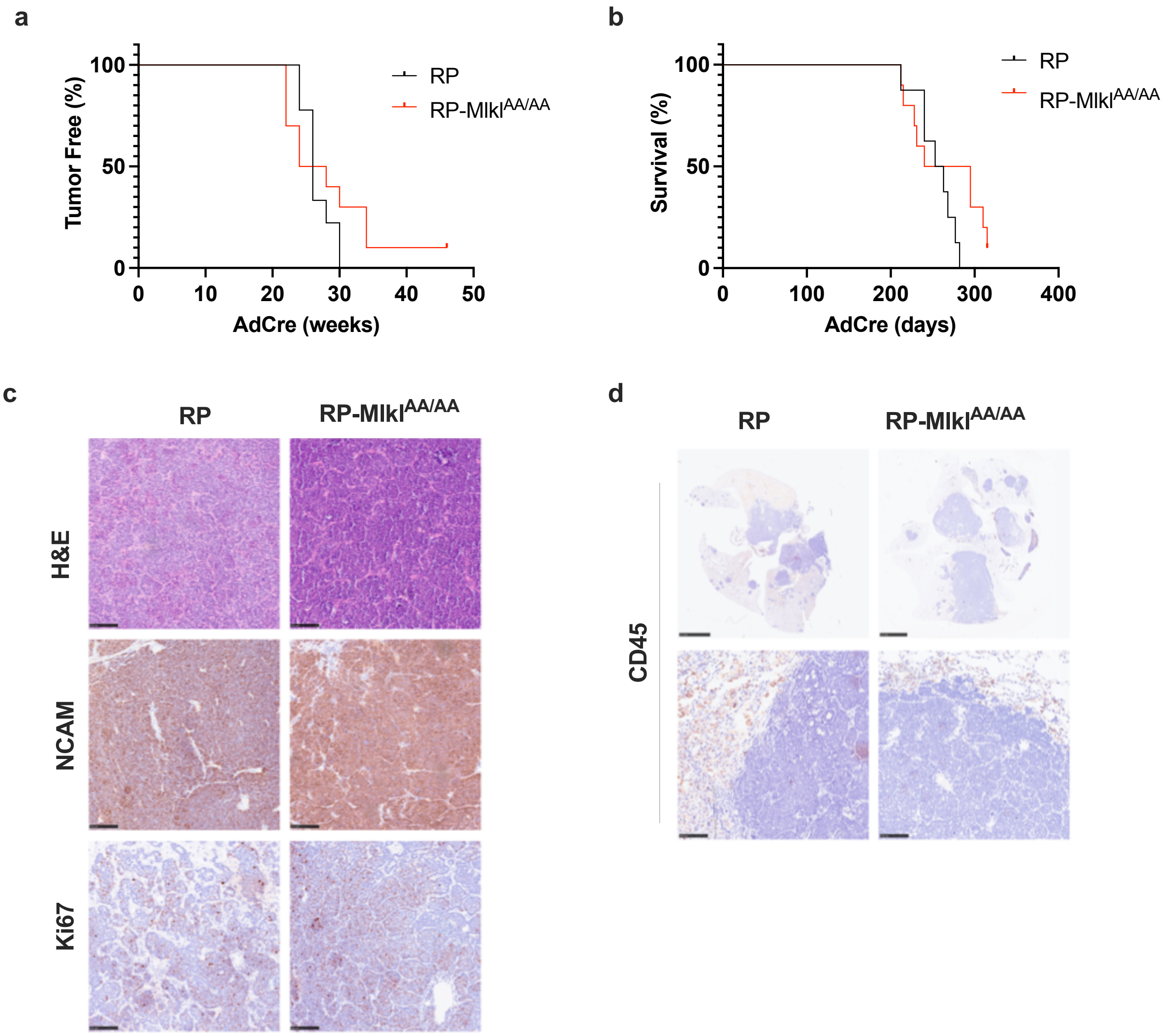


**Supplementary Fig. 11. Constitutive necroptosis does not impact SCLC development in the RP-mouse model.**

**a.** Tumor incidence in RP (n=9) and RP-*Mlkl*$^{AA/AA}$ (n=10) mice after ad-Cre inhalation (AdCre) based on first tumor detection in MRI imaging. p=0.4692 Mantel-Cox test. **b.** Kaplan-Meier survival of RP (n=8) and RP-*Mlkl*$^{AA/AA}$ (n=9). **c.** Representative histological lung images of RP and RP-*Mlkl*$^{AA/AA}$ mice at humane endpoint stained with the indicated antibodies. **d.** samples as in c, stained with the indicated antibody. Source Data are provided as a Source Data file.

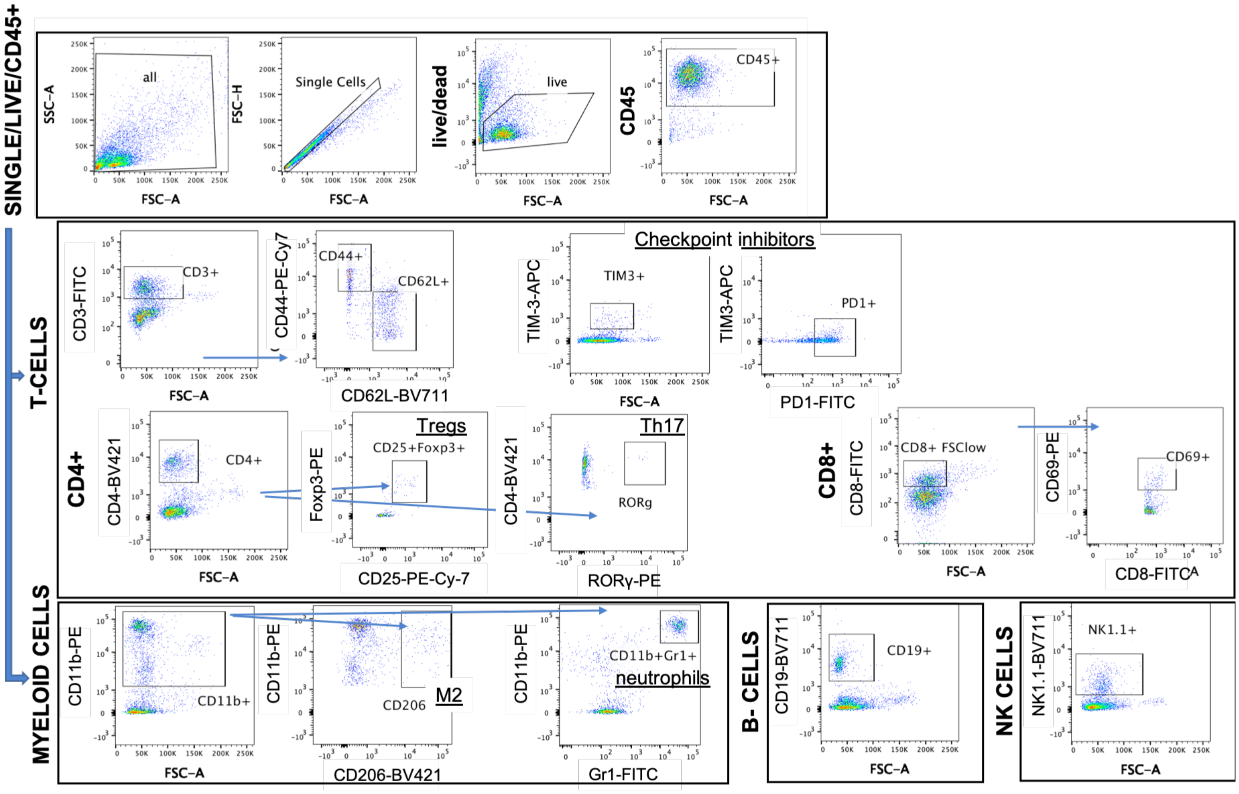


**Supplementary Fig. 12. Flow cytometry gating strategy.**

FACS gating stategy used for the graphs in figures 4c, d, and Sup. fig. 7d-r as well as Sup. fig. 8d. All gates shown for T- cells, Myeloid- cells, B-cells and NK- cells are within single (FSC-H/FSC-A)/ live cells (negative for live/dead dye efluor660 or efluor450).

## Supplementary Note 1

Supplementary Fig. 1b was generated using a screenshot of the CSC Genome Browser[3] showing methylation profiles across the CASP8 gene annotated on hg38. The top tracks display the methylation profiles calculated for each patient separately for normal (blue) and tumor (red) samples, followed by the adjusted p-value and fold-change tracks shown in logarithmic scale (black), and then by summarized tracks for all normal and all tumor samples. The next tracks include gene annotations: RefSeq in collapsed (pack) view[4–6] UCSC annotations showing all CASP8 isoforms for which expression data exist; and GENCODE v44 in collapsed view[7]. The bottom track shows the ENCODE candidate cis-regulatory elements (cCREs), indicating promoter-like signatures (red), proximal enhancer–like signatures (orange), and distal enhancer–

like signatures (yellow) [8–10]. Significantly differentially methylated regions are highlighted in purple, and those located within promoter regions are highlighted in light blue.

**Supplementary References**

1. Q, L. *et al.* Proteogenomic characterization of small cell lung cancer identifies biological insights and subtype-specific therapeutic strategies. *Cell* **187**, (2024).
2. Ireland, A. S. *et al.* MYC Drives Temporal Evolution of Small Cell Lung Cancer Subtypes by Reprogramming Neuroendocrine Fate. *Cancer Cell* (2020).
3. Hinrichs, A. S. *et al.* The UCSC Genome Browser Database: update 2006. *Nucleic Acids Res.* **34**, D590–D598 (2006).
4. Kent, W. J. BLAT--the BLAST-like alignment tool. *Genome Res.* **12**, 656–664 (2002).
5. Pruitt, K. D., Tatusova, T. & Maglott, D. R. NCBI Reference Sequence (RefSeq): a curated non-redundant sequence database of genomes, transcripts and proteins. *Nucleic Acids Res.* **33**, D501-504 (2005).
6. Pruitt, K. D. *et al.* RefSeq: an update on mammalian reference sequences. *Nucleic Acids Res.* **42**, D756-763 (2014).
7. Frankish, A. *et al.* GENCODE: reference annotation for the human and mouse genomes in 2023. *Nucleic Acids Res.* **51**, D942–D949 (2023).
8. ENCODE Project Consortium. An integrated encyclopedia of DNA elements in the human genome. *Nature* **489**, 57–74 (2012).
9. Moore, J. E. *et al.* Expanded encyclopaedias of DNA elements in the human and mouse genomes. *Nature* **583**, 699–710 (2020).
10. A User's Guide to the Encyclopedia of DNA Elements (ENCODE). *PLoS Biol.* **9**, e1001046 (2011).
