# Supplementary material for: Lack of caspase 8 directs neuronal progenitor-like reprogramming and small cell lung cancer progression
Source: Nat Commun. 2025 Dec 18;16:11280. doi: 10.1038/s41467-025-67142-4 (PMC12717127; doi:10.1038/s41467-025-67142-4)
Supplement: Supplementary file 2 — Descriptions of Additional Supplementary Files [file 41467_2025_67142_MOESM2_ESM.pdf]

Descriptions of Additional Supplementary Files

Supplementary Data 1 contains detailed description of the 33 SCLC patient-derived specimens used for Fig. 1d and Supplementary Fig. 1c

Supplementary Data 2 contains the primer sequences of all qPCR primers used in this study
