# Supplementary material for: Lack of caspase 8 directs neuronal progenitor-like reprogramming and small cell lung cancer progression
Source: Nat Commun. 2025 Dec 18;16:11280. doi: 10.1038/s41467-025-67142-4 (PMC12717127; doi:10.1038/s41467-025-67142-4)
Supplement: Supplementary file 5 — Reporting Summary [file 41467_2025_67142_MOESM5_ESM.pdf]

nature portfolio

Corresponding author(s): Silvia von Karstedt

Last updated by author(s): Nov 21, 2025

Reporting Summary

Nature Portfolio wishes to improve the reproducibility of the work that we publish. This form provides structure for consistency and transparency in reporting. For further information on Nature Portfolio policies, see our Editorial Policies and the Editorial Policy Checklist.

Statistics

For all statistical analyses, confirm that the following items are present in the figure legend, table legend, main text, or Methods section.

n/a	Confirmed	
☐	☒	The exact sample size (*n*) for each experimental group/condition, given as a discrete number and unit of measurement
☐	☒	A statement on whether measurements were taken from distinct samples or whether the same sample was measured repeatedly
☐	☒	The statistical test(s) used AND whether they are one- or two-sided<br>*Only common tests should be described solely by name; describe more complex techniques in the Methods section.*
☒	☐	A description of all covariates tested
☐	☒	A description of any assumptions or corrections, such as tests of normality and adjustment for multiple comparisons
☐	☒	A full description of the statistical parameters including central tendency (e.g. means) or other basic estimates (e.g. regression coefficient) AND variation (e.g. standard deviation) or associated estimates of uncertainty (e.g. confidence intervals)
☐	☒	For null hypothesis testing, the test statistic (e.g. *F*, *t*, *r*) with confidence intervals, effect sizes, degrees of freedom and *P* value noted<br>*Give P values as exact values whenever suitable.*
☒	☐	For Bayesian analysis, information on the choice of priors and Markov chain Monte Carlo settings
☒	☐	For hierarchical and complex designs, identification of the appropriate level for tests and full reporting of outcomes
☐	☒	Estimates of effect sizes (e.g. Cohen's *d*, Pearson's *r*), indicating how they were calculated

Our web collection on statistics for biologists contains articles on many of the points above.

Software and code

Policy information about availability of computer code

Data collection	MRI scans were acquired using Philips MR systems Achieva software, FACS data were acquired using BD FACS Diva version 8.0, Live- cell images were acquired with IncuCyte 2022B (Sartorius)
Data analysis	No custom code was used for the study. All published code used for data analysis is described in detail and referenced in the methods section.<br><br>-RNA-sequencing data of patient cohorts were processed with STAR aligner and analyzed with RSEM<br>-Bioinformatics analysis of single-cell RNA sequencing data was done using the PARSE scRNAseq pipeline v.1.1.1. Data analysis was performed using Seurat (v4.4.0)<br>- RNA -seq analysis was conducted using rnaseq pipeline from the nf-core suite (v3.7). STAR aligner (v2.7.10a), DESeq2 (v1.36.0 and fdrtool (v1.2.17) were used for futher analysis as described in the methods part. Heta maps were generated using R.<br>-Methylation sequencing data were were processed in R with QSEA v.1.14.0. Fot he bioinformatics analysis of the methylseq data FastQC v0.11.8, bsmap v2.90, Samtools v1.4, IntersectBed v2.30.0 and ChIPseeker R package were used as described in the methods part.<br>-MRI images were analyzed in Horos v3.3.1 software.<br>-Tissue stainings and tumor spheres were analysed using Omero.web 5.25.0 and BZ-X800 Analyzer software.<br>-qPCR data and ELISA data were analysed using Excel. Graphs and statistical analysis of the data were performed in GraphPad prism v9.5.0<br>-Incucyte data were analyzed using the Software IncuCyte 2022B (Sartorius).<br>-FACS data were analyzed using FlowJo version 10.8.2

For manuscripts utilizing custom algorithms or software that are central to the research but not yet described in published literature, software must be made available to editors and reviewers. We strongly encourage code deposition in a community repository (e.g. GitHub). See the Nature Portfolio guidelines for submitting code & software for further information.

Data

Policy information about availability of data

All manuscripts must include a data availability statement. This statement should provide the following information, where applicable:

- Accession codes, unique identifiers, or web links for publicly available datasets
- A description of any restrictions on data availability
- For clinical datasets or third party data, please ensure that the statement adheres to our policy

Patient derived MeDIP-seq data has been deposited at the European Genome-phenome Archive (EGA), which is hosted by the EBI and the CRG, under accession number EGAS50000000506 (https://ega-archive.org/studies/EGAS50000000506). Further information about EGA can be found at https://ega-archive.org and "The European Genome-phenome Archive of human data consented for biomedical research". Methyl-seq data from RP- and RPC-derived cell lines are available from Gene Expression Omnibus (GEO) under accession number GSE274232 (https://www.ncbi.nlm.nih.gov/geo/query/acc.cgi?acc=GSE274232). Bulk-RNA-seq data from RP- and RPC-derived cell lines are available from GEO, accession number GSE271260 (https://www.ncbi.nlm.nih.gov/geo/query/acc.cgi?acc=GSE271260) and snRNA-seq data from frozen RP- and RPC- mouse tumors are available from GEO, accession number GSE274809 (https://www.ncbi.nlm.nih.gov/geo/query/acc.cgi?acc=GSE274809).

Research involving human participants, their data, or biological material

Policy information about studies with human participants or human data. See also policy information about sex, gender (identity/presentation), and sexual orientation and race, ethnicity and racism.

Reporting on sex and gender	MeDip Sequencing was performed for 33 patient samples previously reported in George et al. Nature 2015. The majority of patients were male (76%) (see Supl. Table 1).
Reporting on race, ethnicity, or other socially relevant groupings	There was no selection of patients based on race, ethnicity, or other socially relevant groupings.
Population characteristics	The median age at the time of first diagnosis was 67 years (referring to 33 patients, Supl. Table 1).
Recruitment	Patients were diagnosed with Small Cell Lung Cancer. No other specific criteria were applied to recruit patients (no clinical study).
Ethics oversight	University of Cologne

Note that full information on the approval of the study protocol must also be provided in the manuscript.

Field-specific reporting

Please select the one below that is the best fit for your research. If you are not sure, read the appropriate sections before making your selection.

☒ Life sciences ☐ Behavioural & social sciences ☐ Ecological, evolutionary & environmental sciences

For a reference copy of the document with all sections, see nature.com/documents/nr-reporting-summary-flat.pdf

Life sciences study design

All studies must disclose on these points even when the disclosure is negative.

Sample size	clinical human samples:<br>MeDip Sequencing was performed for 33 patient samples, previously described in George et al, Nature 2015. Patient samples were chosen based on the availability of high-molecular weight DNA at higher quantities (>3ug) and extracted from lung tumor tissue and adjacent normal lung.<br><br>animal studies:<br>Sample sizes and repeats are indicated in the figure legends/methods and text. Sample sizes were not determined a priori but were chosen based on experience, reproducibility and animal license allowance/limitations.
Data exclusions	No patient was excluded.<br><br>No mouse data exclusions.
Replication	All experiments throughout were performed as biological replicates (n) at least 2-3 times in each cell line. Graphical data are shown as means calculated between these biological replicates +/- standard error mean (SEM). Representative image data are shown from at least two independent biological replicates. All in vivo mouse experiments were repeated in two or three independent cohorts with at least three

independent biological samples in each cohort. The results from all different cohorts were pooled to be shown in figures. No data were excluded from the analyses.

Randomization	clinical human samples (MeDIP-seq samples):<br>Randomization was not applicable in this study, because we describe an exploratory analysis in a discovery cohort.<br><br>Mice were allocated to two groups to obtain an approximately equal number of males and females. When this was not possible, only mice of the same sex were compared between genotypes (either male or female) Where applicable, littermates were allocated to different treatment groups.
Blinding	clinical human samples (MeDIP-seq samples):<br>Blinding was not applicable to this study, because we did not perform a clinical study with a specific clinical question, and instead performed an exploratory analysis in a discovery cohort.<br><br>Investigators were only blinded while performing histological scoring. For the rest, the blinding was not possible as mouse genotype were screened to have necessary selection of mice.

Reporting for specific materials, systems and methods

We require information from authors about some types of materials, experimental systems and methods used in many studies. Here, indicate whether each material, system or method listed is relevant to your study. If you are not sure if a list item applies to your research, read the appropriate section before selecting a response.

Materials & experimental systems

n/a	Involved in the study	
☐	☒	Antibodies
☐	☒	Eukaryotic cell lines
☒	☐	Palaeontology and archaeology
☐	☒	Animals and other organisms
☒	☐	Clinical data
☒	☐	Dual use research of concern
☒	☐	Plants

Methods

n/a	Involved in the study	
☒	☐	ChIP-seq
☐	☒	Flow cytometry
☒	☐	MRI-based neuroimaging

Antibodies

Antibodies used	Western blot:<br>caspase 8, Enzo Life Sciences, ALX-804-447-C100, ß-Actin, Sigma, A1978, a-Tubulin, Santa Cruz, sc-5286 HSP90, Cell Signaling, 4874, Vinculin, Cell Signaling, 13901, ASCL1, BD Pharmingen, 556604, YAP1, Cell Signaling, 4912, REST1, Thermofischer, bs2590, phospho-MLKL(S345), 37333S, MLKL, Millipore MABC604 RIPK1, Cell Signaling 3493, RIPK3, Cell Signaling 15828, phospho-γ2Ax, Cell Signaling 9718<br><br>FACS:<br>CD45-PE (30.F11) and CD45-FITC (30.F11) biolegend, CD4-V450 (RM4-5) BD Horizon, CD25-PE-Cy7 (PC61.5) ebioscience, CD11b-PE (M1/70) ebioscience, CD44-PE-Cy7 (IM7) ebioscience, CD62L-PE (MEL-14) ebioscience, CD3-FITC (145-2C11) ebioscience, CD8-PE (53-6.7) biolegend, CD69-PE (H1.2F3), ebioscience, CD19-BV711 (1D3), BD Horizon, Gr1 (Ly6G/Ly6C) (RB6-BC5), ebioscience, PD1-FITC (J43) ebioscience, TIM3-APC (RMT3-23) ebioscience, NK1.1-FITC (PK-136) biolegend, RORγc-PE (AFKJS-9) ebioscience, CD206-BV421 (C068C2) and Rat IgG2a, κ-isotype Ctrl -BV421 (RTK-2758) biolegend, foxp3-PE (FJK-16s) and Rat IgG2a kappa Isotype Control, PE (eBR2a) ebioscience.<br><br>Histology:<br>anti-cleaved Caspase 3 antibody (Cell Signalling 9661, 1:300) anti-ASCL1 (BD Pharmingen, 556604, 1:500), Ki-67 (Cell Marque 275-R10), CD45 (BD 550539), CD31 (DIA-310-M, Dianova) and CD56 (Zytomed RBK050) and phosho-γ2Ax (Cell Signaling 9718).
Validation	All commercially available antibodies were validated by the supplier

Eukaryotic cell lines

Policy information about cell lines and Sex and Gender in Research

Cell line source(s)	-Mouse SCLC cell lines (AVR 428.1, AVR 404.1G, AVR 132.2) as well as mouse NSCLC cell lines (ACF 132.2, ACF 1035.2 and ACF 135.1) were previously derived from lung tumors of a genetically engineered mouse model for SCLC driven by loss of Trp53 and Rb1 or a genetically engineered mouse model for NSCLC driven by loss of Trp53 and KRASG12D by the lab of Dr. Filippo Beleggia/H. Christian Reinhardt.<br>-RP, RPC, RP-MlklAA/AA and RPC-MlklAA/AA mouse cell lines were newly generated from individual tumors of the respective mice.<br>Human SCLC cell lines (H889, H526 and H82) as well as human NSCLC cell lines (H460, H441 and A549) were obtained from

	ATCC.
Authentication	All human SCLC cell lines where authenticated by STR profiling at Eurofins Genomics.
Mycoplasma contamination	All cell lines were regularly checked for mycoplasma contamination by the mycoplasma tube barcodes service provided by Eurofins Genomics. All cell lines used were confirmed to be mycoplasma negative.
Commonly misidentified lines (See ICLAC register)	None of the cell lines used are commonly misidentified lines.

Animals and other research organisms

Policy information about studies involving animals; ARRIVE guidelines recommended for reporting animal research, and Sex and Gender in Research

Laboratory animals	RbFL/FL mice96 and Tp53FL/FL mice been described previously and were provided be the lab of H. Christian Reinhardt. Casp8FL/FL mice on a C57BL/6 background were obtained under a material transfer agreement (MTA) from Stephen Hedrick. MlklAA/AA mice were newly generated in the Pasparakis lab and described in Körner et al.
Wild animals	n/a
Reporting on sex	Animals of both sexes were used in all experiments
Field-collected samples	n/a
Ethics oversight	All animal experiments were approved by the local authorities (LANUV, North-Rhine-Westphalia, Germany). All people involved in animal experiments received prior training and have passed the additionally required personal licensing course (FELASAB).

Note that full information on the approval of the study protocol must also be provided in the manuscript.

Plants

Seed stocks	n/a
Novel plant genotypes	n/a
Authentication	n/a

Flow Cytometry

Plots

Confirm that:

☒ The axis labels state the marker and fluorochrome used (e.g. CD4-FITC).

☒ The axis scales are clearly visible. Include numbers along axes only for bottom left plot of group (a 'group' is an analysis of identical markers).

☒ All plots are contour plots with outliers or pseudocolor plots.

☒ A numerical value for number of cells or percentage (with statistics) is provided.

Methodology

Sample preparation	Single cell suspensions of immune cells obtained from tumors were stained for live-dead cells, washed, incubated with Fc block and stained for surface markers. For intracellular staining, the eBioscience™ Foxp3/transcription factor staining buffer kit was used. In this case, after surface marker staining, cells were fixed and permeabilized, blocked with goat serum and stained intracellularly.
Instrument	FACS data were acquired on an LSR Fortessa (cat no. 647788, BS Bioscience), November 2013 model no. 647788E3

Software	Data were acquired using Diva software (BD Bioscience), analysis was performed using FlowJo version 10.8.2 or Diva (8.0)
Cell population abundance	10000-20000 cells were acquired to measure abundance of target cell population.
Gating strategy	cells were gated (FSC.A/SSC.A)=> singlets (FSC.A/FSC.H), live cells (fixable live/dead dye+), CD45+ cells . Stainings for specific populations followed as described in Supplementary Fig. 12

☒ Tick this box to confirm that a figure exemplifying the gating strategy is provided in the Supplementary Information.
